# The open architecture of HD-PTP phosphatase provides new insights into the mechanism of regulation of ESCRT function

**DOI:** 10.1038/s41598-017-09467-9

**Published:** 2017-08-22

**Authors:** Deepankar Gahloth, Graham Heaven, Thomas A. Jowitt, A. Paul Mould, Jordi Bella, Clair Baldock, Philip Woodman, Lydia Tabernero

**Affiliations:** 1 School of Biological Sciences, Faculty of Biology Medicine and Health, University of Manchester, Manchester Academic Health Science Centre, Manchester, UK; 2 School of Chemistry and Photon Science Institute, University of Manchester, Manchester, UK; 3 Biomolecular Analysis Core Facility, Faculty of Biology Medicine and Health, University of Manchester, Manchester Academic Health Science Centre, Manchester, UK

## Abstract

HD-PTP is a tumour suppressor phosphatase that controls endocytosis, down-regulation of mitogenic receptors and cell migration. Central to its role is the specific recruitment of critical endosomal sorting complexes required for transport (ESCRTs). However, the molecular mechanisms that enable HD-PTP to regulate ESCRT function are unknown. We have characterised the molecular architecture of the entire ESCRT binding region of HD-PTP using small angle X-ray scattering and hydrodynamic analyses. We show that HD-PTP adopts an open and extended conformation, optimal for concomitant interactions with multiple ESCRTs, which contrasts with the compact conformation of the related ESCRT regulator Alix. We demonstrate that the HD-PTP open conformation is functionally competent for binding cellular protein partners. Our analyses rationalise the functional cooperation of HD-PTP with ESCRT-0, ESCRT-I and ESCRT-III and support a model for regulation of ESCRT function by displacement of ESCRT subunits, which is crucial in determining the fate of ubiquitinated cargo.

## Introduction

His Domain Protein Tyrosine Phosphatase (HD-PTP) is essential for the lysosomal degradation of multiple membrane receptors, including activated EGFR and PDGFR-β^1, 2^, MHC Class I^3^ and α5β1 integrin^4^. This process requires sorting of the ubiquitinated receptors to intralumenal vesicles (ILVs) within the mutivesicular body (MVB), and their subsequent delivery to the lysosome^5^. Central to HD-PTP function during MVB sorting is the binding to specific endosomal sorting complexes required for transport (ESCRTs)^1, 6, 7^. However, little is known at the molecular level about how HD-PTP regulates ESCRT function.

ESCRTs are multimeric protein complexes (numbered 0, I, II, III) that control many membrane remodelling and scission events critical to cell physiology^8, 9^. These events include ILV formation at the MVB^5^, midbody abscission during cytokinesis^10, 11^, exosome formation^12^, autophagy^13^, plasma membrane wound repair^14^, nuclear envelope remodelling^15, 16^ and neuron pruning^17, 18^. Viruses also exploit the ESCRT machinery to facilitate virion budding^19^.

Different subsets of ESCRTs and specialised adaptor proteins define pathway selectivity but they all culminate in the assembly of membrane sculpting ESCRT-III polymers at the point of membrane scission^8, 9, 20, 21^. The mechanisms that regulate the different ESCRT pathways are still poorly understood and deciphering how ESCRT-III assembly is ultimately coordinated at each location remains an open question. HD-PTP and the related ESCRT adaptor protein, Alix, are central to such functional specialisation. Alix directly regulates ESCRT-III recruitment and assembly at a range of sites^10, 11, 21–23^. In contrast, HD-PTP function is largely restricted to MVB biogenesis^1, 3^.

**Figure 1:**
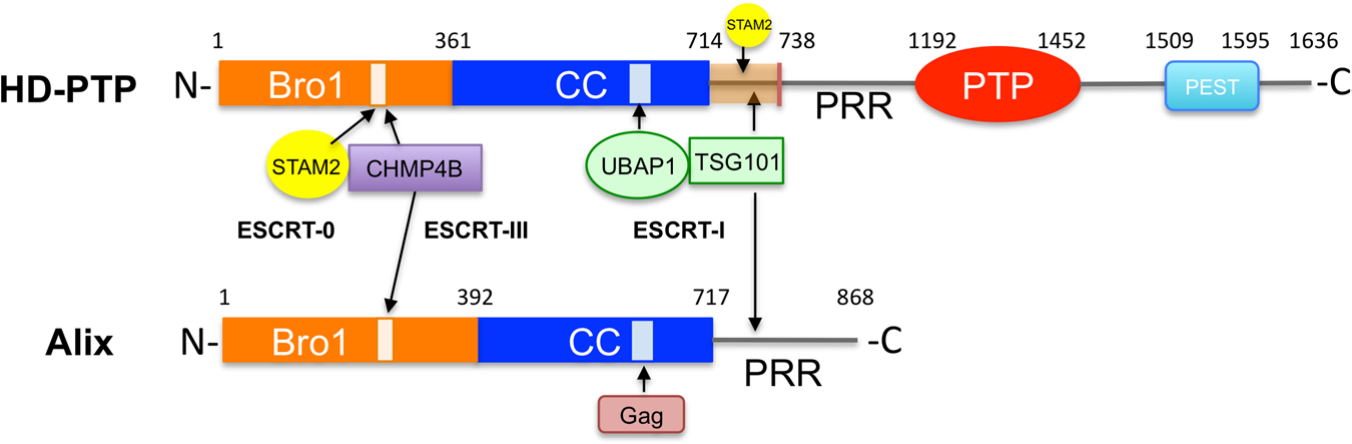
Domain organisation of HD-PTP and Alix. Residue numbers indicate domain boundaries and arrows show locations of binding for the different interacting partners. Domain names: Bro1; CC, coiled-coil; PRR, proline rich region; PTP, protein tyrosine phosphatase; PEST, rich in proline (P), glutamate (E), serine (S) and threonine (T). The proximal region of PRR in HD-PTP is indicated as a brown box (residues 714–738).

HD-PTP has a multidomain organisation^24^ (Fig. 1), with an N-terminal Bro1 domain that binds ESCRT-0/STAM2 and ESCRT-III/CHMP4^7, 25–28^, followed by a coiled-coil domain (CC) that interacts with the ESCRT-I subunit UBAP1^6, 29^, a proline-rich region (PRR) with binding sites for the ESCRT-I subunit TSG101^27^ as well as a further site for STAM2, a protein tyrosine phosphatase (PTP) domain, and a C-terminal PEST domain. Alix shares a similar Bro1-CC-PRR organisation, including binding sites for CHMP4 and TSG101, but lacks the PTP and PEST domains (Fig. 1, Suppl. Fig. S1).

Alix function is highly regulated and it exists in the cytoplasm as an inactive form that is prevented from binding to ESCRT-III and viral Gag protein substrates^30, 31^. Self-inhibition is favoured by the compact architecture of the Bro1-CC region, where the CC domain adopts a closed V shape^32^ that brings the proximal region of the PRR to interact with the Bro1 domain^33^. This arrangement blocks access of CHMP4 and Gag proteins to their binding sites on the Alix surface. Alix activation occurs by several mechanisms including phosphorylation^34^, binding of the membrane adaptors CEP55^35^ and ALG-2^34^ to the PRR, and subsequent conformational rearrangements and dimerisation^36, 37^.

In contrast to Alix, nothing is known about the regulation of HD-PTP interaction with ESCRTs at the structural level, and particularly, whether a similar self-inhibition mechanism applies. We have previously reported that STAM2 competes with CHMP4 for binding to the Bro1 domain of HD-PTP, but does not bind to Alix^7^. STAM2 also binds to the HD-PTP PRR at a site that overlaps with the TSG101^7^ (Fig. 1). In addition, UBAP1 binds to the CC domain of HD-PTP in a selective manner^6, 29^. Our structural analyses of HD-PTP in complex with different ESCRT subunits and endosomal effectors have shown that both amino acid sequence and molecular determinants are key to define the specificity and selectivity observed for these interactions^25, 29^. Altogether these data highlight that coupling between the Bro1, CC and proximal PRR regions of HD-PTP may control the exchange of ESCRT partners, which is essential for HD-PTP function. The importance of the Bro1-CC region of HD-PTP is underscored by findings that it represents the minimal functional region of HD-PTP^1^.

Knowledge of the molecular architecture of HD-PTP is thus critical for understanding the mechanism of regulation of its interactions with ESCRTs. Whilst high-resolution structures of the Bro1 domain^25, 38, 39^ and the CC domain^29^ have been reported, the structural coupling of these two domains remains unexplored. Here, we present a structural and biophysical analysis of the entire ESCRT-binding region of HD-PTP (encompassing the Bro1 and CC domains and the proximal region of PRR) by small angle X-ray scattering (SAXS), analytical ultracentrifugation (AUC), size-exclusion chromatography (SEC), and multi-angle light-scattering (MALS). The structures of HD-PTP fragments encompassing the CC domain (HD-PTP_CC_), the Bro1 and CC domains (HD-PTP_Bro1-CC_), and the Bro1 and CC domains plus the proximal region of PRR (HD-PTP_Bro1-CC-PRR_) all show an open and extended conformation where the Bro1 and CC domains are spread out horizontally, thus providing a large scaffolding architecture. Hydrodynamic parameters obtained from AUC and SEC-MALS confirmed the extended conformation and the monomeric nature of all three proteins. The open architecture of HD-PTP offers an ideal platform for binding of multiple ESCRTs to the Bro1, CC and PRR domains, and suggests a mechanism of regulation by which displacement of ESCRT subunits controls access to ESCRT-III.

## Results

### The extended architecture of HD-PTP_CC_, HD-PTP_Bro1-CC_ and HD-PTP_Bro1-CC-PRR_ rules out a self-inhibited conformation

**Figure 2:**
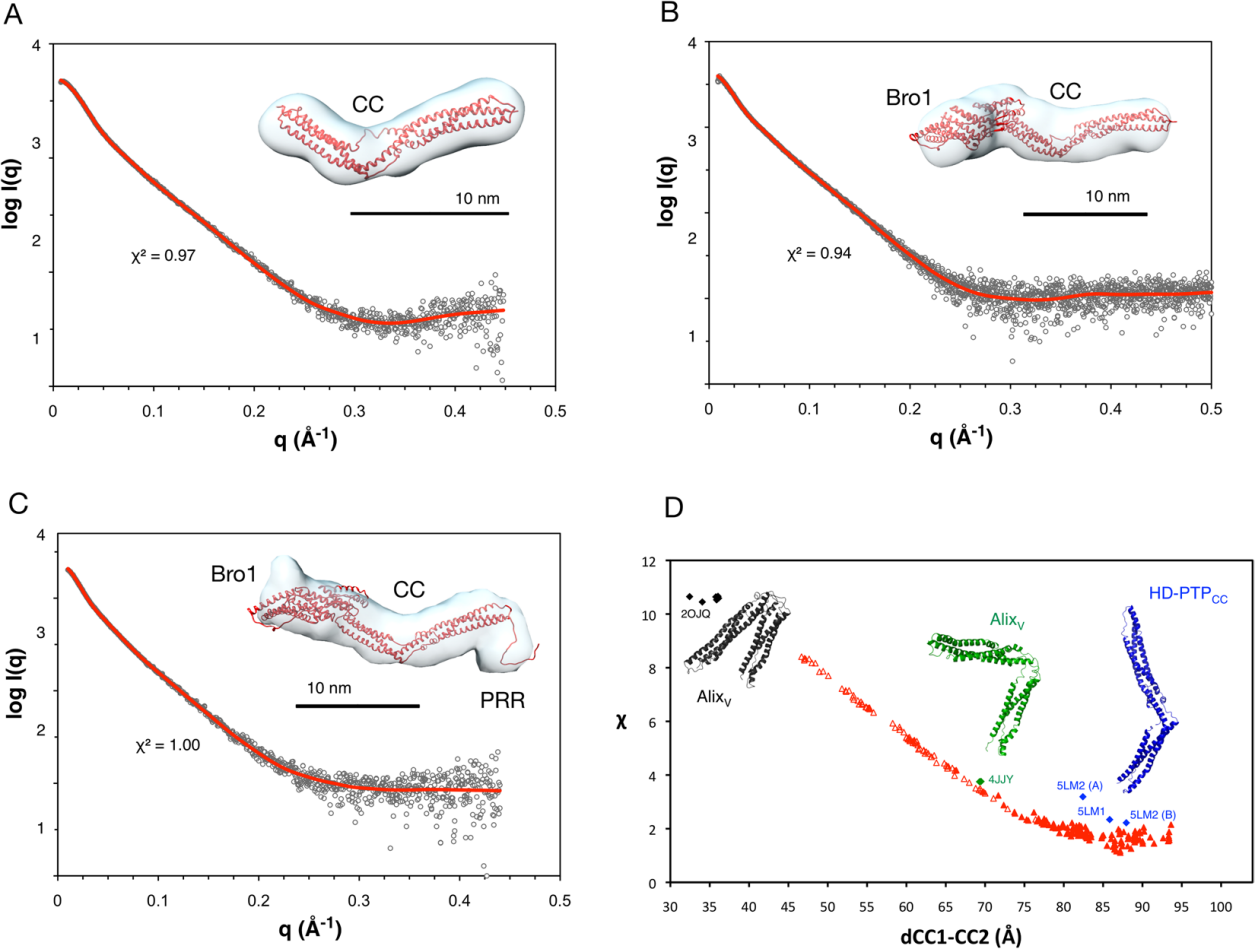
Analysis of HD-PTP by Small Angle X-ray Scattering. (**A**–**C**) Plots of X-ray scattering intensity log(I(q)) as a function of the scattering vector q (Å^−1^) for HD-PTP_CC_ **(A**), HD-PTP_Bro1-CC_ (**B**) and HD-PTP_Bro1-CC-PRR_ (**C**). SAXS data are shown as grey open circles and the normalised fit from the best individual molecular model is shown as a red line (calculated with FoXS^55^, goodness of fit indicated with its χ^2^ value). *Ab initio* reconstructions calculated with DAMAVER^52^ are shown as transparent envelopes with the best individual models superimposed using the automatic map fitting tool in Chimera^58^. The N-terminal tails of HD-PTP_Bro1-CC_ and HD-PTP_Bro1-CC-PRR_ are not shown, for clarity. (**D**) Variation of the goodness of fit (χ) with the HD-PTP_CC_ SAXS data as a function of the distance between the centre of mass of subdomains CC1 and CC2 (dCC1-CC2). Fits from crystal structure coordinates of HD-PTP_CC_ (blue) and Alix_V_ (grey, closed forms; green, open forms) are individually shown (selected PDB ID codes indicated). Fits from HD-PTP_CC_ models with varying CC1-CC2 distances are shown as red triangles. Filled triangles correspond to models with calculated sedimentation coefficients (SOMO^45^) consistent with the experimentally observed value (Table 1). Best χ values and agreement with hydrodynamic data are observed for the extended forms.

Crystal structures of Alix_Bro1-V_ have shown a compact conformation in which the Bro1 and V domains are packed against each other. In addition, the two arms of the V domain (V1 and V2) form a bent, rather than extended molecular shape^32^. Further studies indicated that the PRR region binds to the Bro1 domain, locking Alix in a functionally inactive conformation^33, 34^. We have recently reported that the CC domain of HD-PTP adopts an extended open conformation in the crystallographic structure^29^, quite distinct to the closed V-shaped form of the equivalent domain in Alix^32^. To gain insight into the architecture of the Bro1-CC region and to assess if HD-PTP undergoes similar regulation as Alix, we investigated the molecular shape and dimensions of HD-PTP in solution by collecting small-angle X-ray scattering (SAXS) measurements on HD-PTP_CC_, HD-PTP_Bro1-CC_, and HD-PTP_Bro1-CC-PRR_ (Fig. 2).

Analysis of the dimensionless Kratky plots indicates that none of the three proteins are globular as the peak maxima do not coincide with the globularity point, and the shapes of the curves suggest that they are folded, elongated proteins (Suppl. Fig. S2). Porod exponents also confirm they are compact molecules (Table 1). The Porod-Debye plots (q^4^ × I(q) against q^4^) and SIBYLS plots (q^3^ × I(q) against q^3^) are consistent with compact particles^40^ and only the HD-PTP_Bro1-CC-PRR_ construct shows some sign of limited flexibility due to the presence of the unstructured PRR region.

**Table 1:** Hydrodynamic and dimensional data for HD-PTP.

*Experimental*	MALS			AUC				SAXS	
	*R*_*h*_ (nm)	M_r_ (Da) exp./calc.	*R*_*h*_ (nm)	*f/f*_*0*_	*S*_20,W_	*R*_*g*_ (nm)	*D*_*max*_ (nm)	*P*	*V* (*A* ^3^)
HD-PTP_CC (no tag)_	3.93 ± 0.12	38,200/38,987	4.07	1.76	2.47 ± 0.19	4.53	15.3	2.8	67,664
HD-PTP_Bro1-CC (+tag)_	4.97 ± 0.12	85,900/83,459	5.04	1.74	3.86 ± 0.10	5.55	19.3	3.3	123,506
HD-PTP_Bro1-CC-PRR (+tag)_	5.47 ± 0.05	87,900/86,107	5.52	1.88	3.63 ± 0.13	5.83	20.4	3.3	126,320
***Calculated*** **(SOMO)**	**Coordinates**		***R***_***h***_ **(nm)**	***f/f***_**0**_	***S***_**20,W**_	***R***_***g***_ **(nm)**	***D***_***max***_ **(nm)**		
HD-PTP_CC_	5LM2		3.62	1.61	2.60	4.47	16.3		
HD-PTP_CC_	model		3.66	1.63	2.50	4.37	16.1		
HD-PTP_Bro1-CC_	model		5.26	1.81	3.71	6.10	22.0		
HD-PTP_Bro1-CC-PRR_	model		5.51	1.88	3.64	6.39	25.0		
Alix_V_	4JJY		3.61	1.61	2.52	3.88	13.2		
Alix_V_	2OJQ		3.22	1.44	2.83	2.91	11.2		
Alix_Bro1-V_	2OEV		4.32	1.52	4.16	4.49	16.5		

Experimental parameters were determined from MALS, AUC and SAXS measurements. Hydrodynamic parameters for the crystal structures of HD-PTP_CC_^29^, Alix_V_^47^ and Alix_Bro1-V_^32^ and the best SAXS models of HD-PTP_CC_, HD-PTP_Bro1-CC_ and HD-PTP_Bro1-CC-PRR_ were calculated with SOMO^45^. *R*_*h*_ hydrodynamic radius; *S*_20,w_ sedimentation coefficient in water at 20 °C; *f/f*_0_ frictional coefficient; *R*_*g*_ radius of gyration; *D*_*max*_, maximal linear dimension of the particles; *P*, Porod exponent; *V*, Porod volume.

Estimates of the radius of gyration (*R*_*g*_) for each protein were obtained from the Guinier region using PRIMUS^41^, and the maximum dimension (*D*_*max*_) was obtained from indirect Fourier transformation of the SAXS profiles using GNOM^42^ (Table 1, Suppl. Fig. S3). For HD-PTP_CC_ the *D*_*max*_ of 15.3 nm is similar to that measured in its crystal structure^29^. For HD-PTP_Bro1-CC_ and HD-PTP_Bro1-CC-PRR_ the *D*_*max*_ obtained were 19.3 nm and 20.4 nm respectively (Table 1). The probable atom-pair distribution functions *p*(*r*) for the three proteins (Suppl. Fig. S3) present asymmetrical curves consistent with elongated molecules. Particle shapes were restored *ab initio* from the SAXS profiles using DAMMIN and GASBOR^43, 44^, and they were all consistent with an open and extended molecular architecture (Fig. 2). The particle shape of HD-PTP_Bro1-CC-PRR_ shows a visible extension at the C-terminal end of the CC domain, corresponding to the additional residues in the PRR region (Fig. 2c).

Molecular models for each construct of HD-PTP were produced to help in the interpretation of the SAXS data (see Methods). The HD-PTP_CC_ crystal structure coordinates^29^ show relatively poor fits to the SAXS profile (Fig. 2d). Coordinates of Alix_V_ domains obtained from Alix crystal structures (open and closed forms) give even poorer fits (Fig. 2d). We therefore generated a library of HD-PTP_CC_ conformers where the angle between the “blade” (CC1) and “shaft” (CC2) subdomains^29^ was changed by variation of the distance d(CC1-CC2) between their centre of masses (see Methods for details). In general, extended conformations with d(CC1-CC2) of 85–90 Å fit the SAXS data much better than closed conformations (Fig. 2d). The best individual model (χ^2^ = 0.98, Fig. 2a) shows a d(CC1-CC2) of 88 Å.

Molecular models for HD-PTP_Bro1-CC_ were generated using the published structures of the Bro1 domain^38^ and the CC domain^29^. Models for HD-PTP_Bro1-CC-PRR_ contained the additional residues (714–738) corresponding to the proximal region of the PRR. A library of conformers was generated using torsion angle molecular dynamics (TAMD) by varying the orientation between the Bro1 and CC domains (see Methods). Hydrodynamic parameters for each HD-PTP_Bro1-CC_ conformer were calculated using SOMO^45^ and compared with the experimentally determined values. A subset of conformers was selected based on their sedimentation coefficients values, as in ref. 46. The best individual models (HD-PTP_Bro1-CC_ χ^2^ = 1.00; HD-PTP_Bro1-CC-PRR_ χ^2^ = 0.96) show an extended linear arrangement between the Bro1 and CC domains (Fig. 2b,c). Thus, HD-PTP_Bro1-CC_ and HD-PTP_Bro1-CC-PRR_ have very similar conformation in solution and there is no evidence of a compact conformation induced by the addition of the PRR region, as reported for Alix^30–33^.

### Hydrodynamic analyses confirm HD-PTP is a monomer with an extended conformation

**Figure 3:**
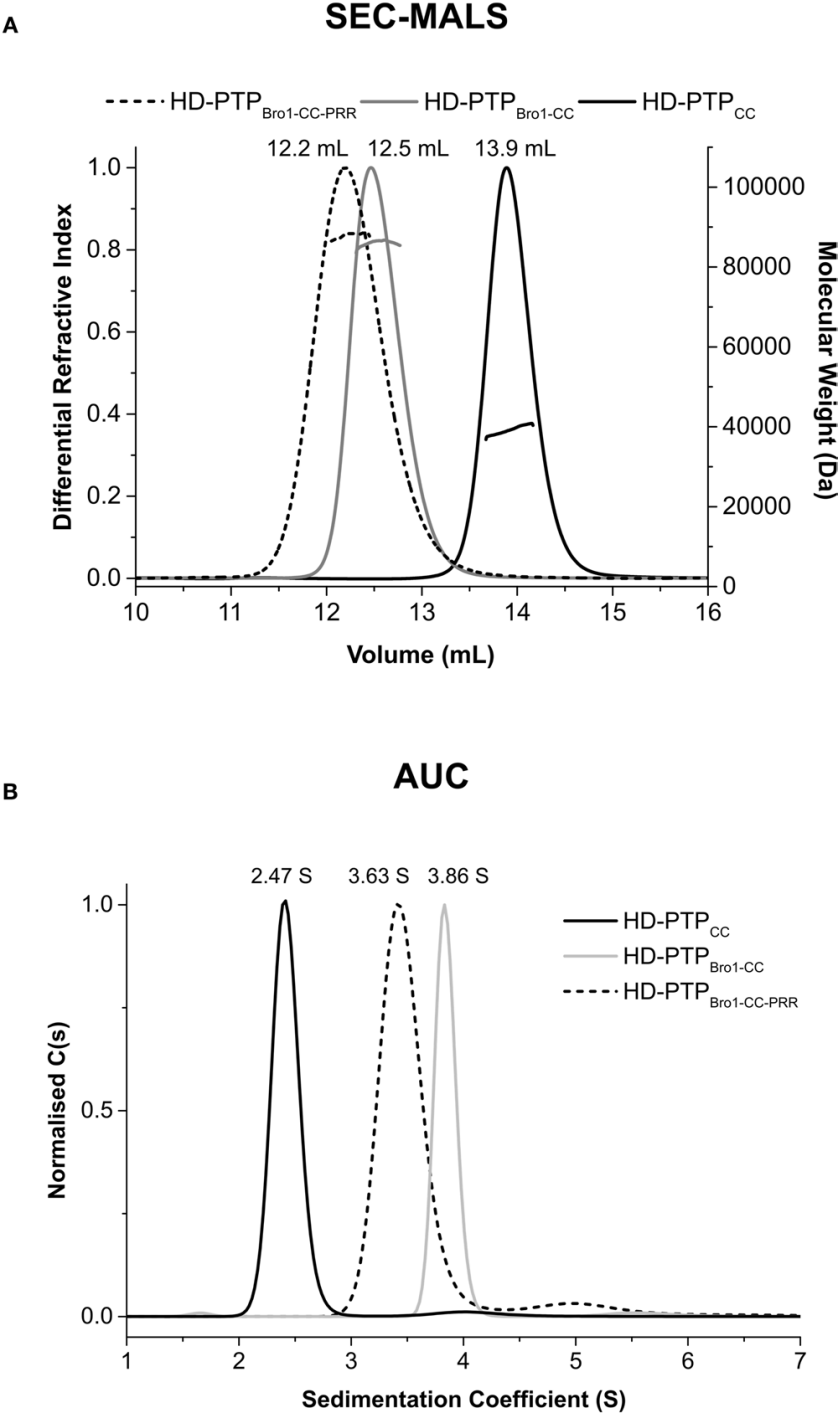
Hydrodynamic analyses of HD-PTP. (**A**) SEC-MALS profile of HD-PTP_CC_, HD-PTP_Bro1-CC_ and HD-PTP_Bro1-CC-PRR_. The chromatogram shows differential refractive index (normalised) and molecular weight versus elution volume. The elution profile shows single peaks indicating monodisperse samples. The average molecular weights of 38 kDa for HD-PTP_CC_, 86 kDa for HD-PTP_Bro1-CC_ and 88 for HD-PTP_Bro1-CC-PRR_ indicate monomeric particles for each protein. (**B**) AUC- derived sedimentation coefficient distributions for HD-PTP_CC_, HD-PTP_Bro1-CC_ and HD-PTP_Bro1-CC-PRR_ show single peaks for each construct, labelled with the corresponding buffer-corrected *S*_20,w_ values. Other hydrodynamic parameters are shown in Table 1.

Hydrodynamic parameters were determined using SEC-MALS and AUC (Fig. 3, Table 1). All three proteins behaved as monomers according to their elution profile (Fig. 3A), with average molecular weights of 38 kDa for HD-PTP_CC_, 86 kDa for HD-PTP_Bro1-CC_ and 88 kDa for HD-PTP_Bro1-CC-PRR_. Hence, in contrast to Alix^36, 37^, the HD-PTP constructs tested appear to lack an intrinsic ability to dimerise in solution. Furthermore, sedimentation velocity experiments showed single species for each protein with sedimentation coefficients of 2.47 S for HD-PTP_CC,_ 3.86 S for HD-PTP_Bro1-CC_ and 3.63 S for HD-PTP_Bro1-CC-PRR_ (Fig. 3B, Table 1). The decrease in the sedimentation coefficient upon addition of the PRR indicates an increase in asymmetry of the particle and is consistent with our SAXS model and larger *D*_*max*_.

**Figure 4:**
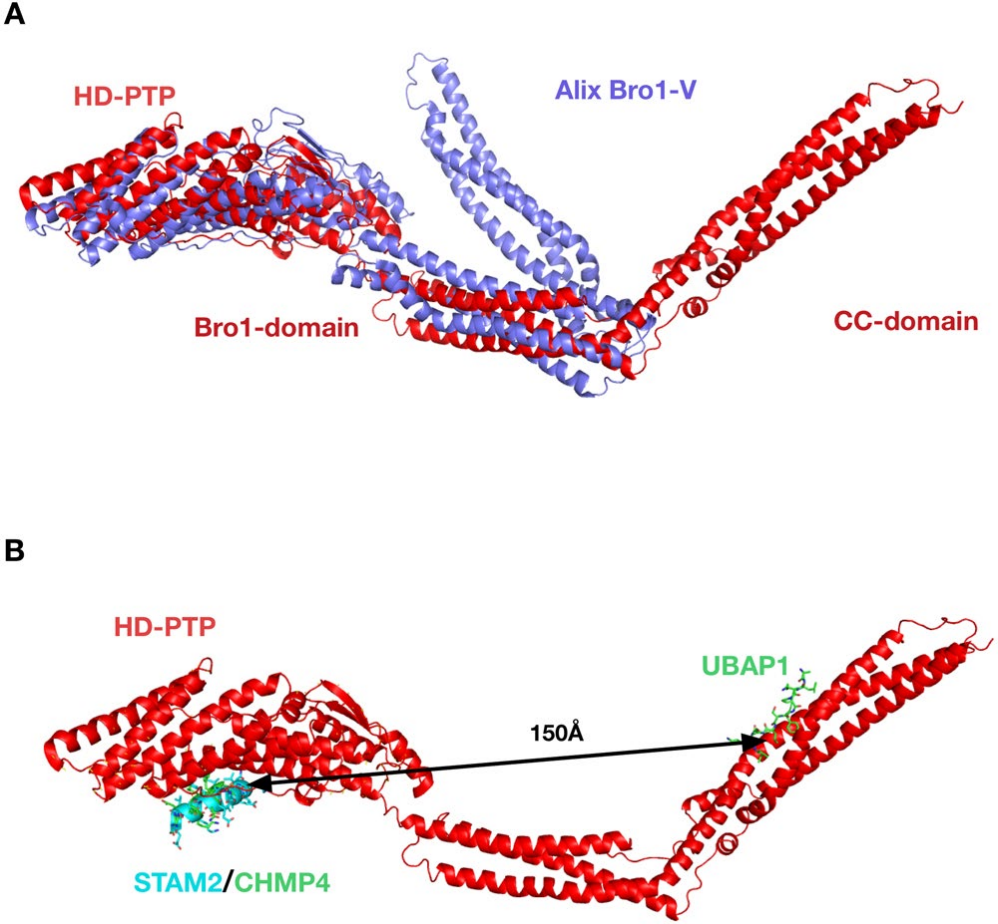
The molecular shapes of HD-PTP and Alix differ. (**A**) Superimposition of the best individual model of HD-PTP_Bro1-CC_ (red) with the crystal structure of Alix_Bro1-V_ (purple) (PDB ID 2OEV) showing the contrast between the open and extended structure of HD-PTP versus the more compact conformation of Alix. **(B)** Ribbon diagram of HD-PTP_Bro1-CC_ with superimposed models of the ESCRT subunits STAM2 (cyan), CHMP4B (green) and UBAP1 (green) bound to their respective binding sites^25, 29, 39^. The arrow shows the approximate distance between binding sites.

The hydrodynamic radius and frictional ratio of HD-PTP_CC_ (*R*_*h*_ = 4.07 nm, *f/f*_0_ = 1.76) confirmed an anisotropic and elongated shape consistent with that observed in its crystal structure^29^. Hydrodynamically, HD-PTP_CC_ is clearly different from the V-shaped structure of Alix_V_^47^ (*R*_*h*_ = 3.22 nm, *f/f*_0_ = 1.44). Likewise, both HD-PTP_Bro1-CC_ and HD-PTP_Bro1-CC-PRR_ adopted elongated conformations (*R*_*h*_ = 5.04 nm, *f/f*_0_ = 1.74 and R_*h*_ = 5.52 nm, *f/f*_0_ = 1.88, respectively) that contrast with the compact structure of Alix_Bro1-V_^32^ (*R*_*h*_ = 4.32 nm and *f/f*_0_ = 1.52) (Fig. 4, Table 1). The addition of the PRR region significantly increases the asymmetry of the molecule as shown by the decrease in sedimentation coefficient (above) and subsequent increase in the frictional ratio. Together these data demonstrate that, unlike Alix, HD-PTP exists in solution as an open and extended molecule, with no evidence for a closed, self-inhibited conformation, even in the presence of a functionally important region of the PRR.

### The HD-PTP open conformation is functionally active

**Figure 5:**
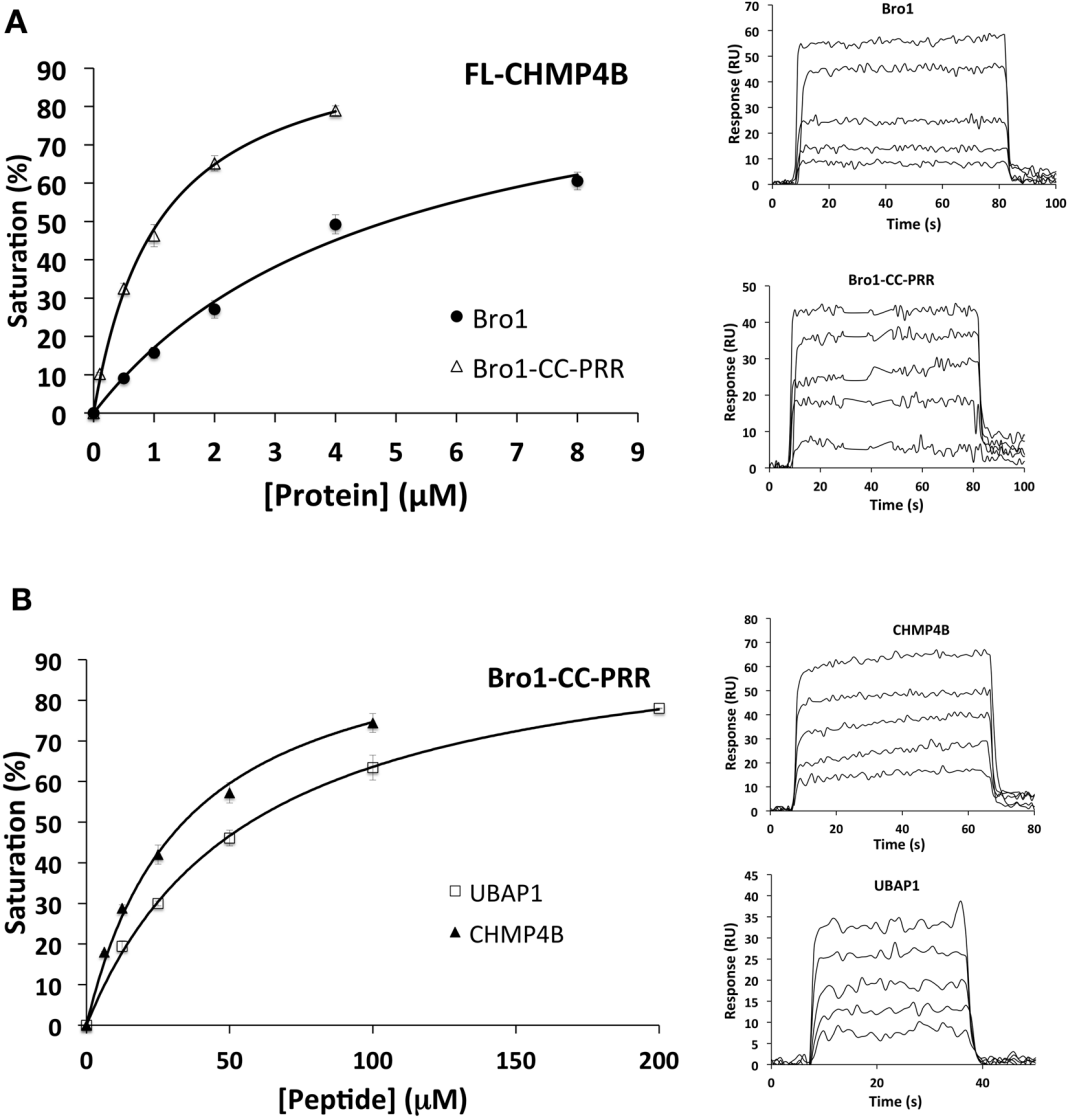
Analysis of HD-PTP binding to ESCRT subunits. (**A**) Equilibrium biosensor binding analysis for immobilised full length CHMP4B to HD-PTP_Bro1_ (*K*_*d*_ 4.88 ± 0.3 μM) and HD-PTP_Bro1-CC-PRR_ (*K*_*d*_ 1.1 ± 0.1 μM), Biosensor sensograms shown in the insets. **(B)** Equilibrium biosensor binding analysis for immobilised HD-PTP_Bro1-CC-PRR_ to CHMP4B peptide (*K*_*d*_ 30.9 ± 0.2 μM) and to UBAP1 peptide (*K*_*d*_ 57.5 ± 3.1 μM). Biosensor sensograms shown in the insets.

We have previously reported that HD-PTP_Bro1-CC_ is the minimal functional region, able to rescue defects in endosomal trafficking of ubiquitinated cell-surface receptors^1^, and also sufficient for UBAP1 binding in a cellular setting^29^. Here we show that in contrast to Alix_Bro1-V-PRR_^30, 31^, HD-PTP_Bro1-CC-PRR_ is fully functional for binding to ESCRT partners. We used biosensor binding experiments (SPR) to demonstrate that both HD-PTP_Bro1_ and HD-PTP_Bro1-CC-PRR_ are able to bind full length CHMP4B (FL-CHMP4B) with similar affinity (Fig. 5A). HD-PTP_Bro1-CC-PRR_ also binds peptides from the C-terminus of CHMP4B (residues 205–224) and the central region of UBAP1 (residues 261–280) (Fig. 5B) with similar affinity to that observed for the individual Bro1 or CC domains respectively^29^. These results indicate that each binding site is fully accessible in HD-PTP_Bro1-CC-PRR_ and that the architecture observed in solution is compatible with ESCRT binding and remains functionally active in the presence of the PRR proximal region.

## Discussion

Our findings provide significant new insights into how HD-PTP controls the ESCRT pathway at the endosome, behaving differently from the canonical Bro1 domain -containing protein Alix. First, they show that HD-PTP_Bro1-CC_ offers a far more extended ESCRT binding platform than Alix_Bro1-V_ (Fig. 4A). Indeed, the open architecture of HD-PTP is compatible with binding to multiple ESCRT partners, allowing it to act as a hub for the traffic of endocytic cargo along the ESCRT pathway. Second, our findings show that the mechanisms of self-inhibition elucidated for Alix do not apply to HD-PTP. Together these results invoke a new mechanism of ESCRT regulation, by which HD-PTP could act as central orchestrator to determine whether ubiquitinated cargoes are degraded, recycled, or held at the endosome, in keeping with its function as a tumour suppressor.

Our data shows that HD-PTP_Bro1-CC_ adopts an open and extended architecture that is also maintained in HD-PTP_Bro1-CC-PRR_, a construct including the proximal region of PRR. The CC domain maintains the extended conformation observed in its crystallographic structure^29^, and the Bro1 domain provides a largely linear extension to the molecule, with limited flexibility between the two domains (Suppl. Fig. S2). This extended conformation is functionally competent, as both HD-PTP_Bro1_ and HD-PTP_Bro1-CC-PRR_ are able to bind full length CHMP4B and HD-PTP_Bro1-CC-PRR_ also binds to the central region of UBAP1, as demonstrated by biosensor binding experiments (Fig. 5 and ref. 29). We have previously demonstrated the ability of HD-PTP_Bro1-CC_ to bind UBAP1 in cells and to rescue defects in EGFR sorting^1, 29^.

Such an extended architecture is consistent with the proposed role for HD-PTP as a scaffold platform for ESCRT coordination^1, 6, 7^. In particular, the long distance between the CHMP4B binding site in the Bro1 domain and the UBAP1 site in the CC-domain (~150 Å, Fig. 4B) indicates that HD-PTP would be able to interact simultaneously with ESCRT-I and ESCRT-III. The ability to link ESCRT-I with ESCRT-III may be especially relevant to the reported essential requirement of HD-PTP, but not Alix, in promoting cargo trafficking in the absence of ESCRT-II^3, 18^.

Alix is subject to auto-inhibition, adopting a closed conformation that prevents effector binding to the Bro1 and V domains and undergoes large-scale structural rearrangements and dimerisation upon activation^32, 33, 37^. In contrast, we find no evidence for dimerisation, large-scale rearrangements, or the presence of closed self-inhibited forms in solution of HD-PTP. Furthermore, the presence of the proximal region of PRR does not alter the open architecture of HD-PTP and, crucially, does not preclude binding of HD-PTP to critical ESCRT subunits including CHMP4B and UBAP1.

Since HD-PTP_Bro1-CC_ lacks the conformational variability of Alix, alternative mechanisms of regulating access to ESCRT-III must come into play. An attractive hypothesis is that interactions of both ESCRT-I and ESCRT–III with HD-PTP are controlled by ESCRT-0, consistent with the role of ESCRT-0 in deciding whether ubiquitinated cargo is recycled or sorted into the MVB pathway^48^. In support of this idea is the evidence that the ESCRT-0 subunit STAM2 binds to the Bro1 domain of HD-PTP at the conserved CHMP4B binding region^7, 39^, and also binds to the proximal region of PRR at a consensus SH3 binding motif (PPRPTAPKP) that includes the binding site for TSG101 (Suppl. Fig. 1)^7^. It is therefore possible that ESCRT-0 (which forms an extended dimer) binds both sites simultaneously.

**Figure 6:**
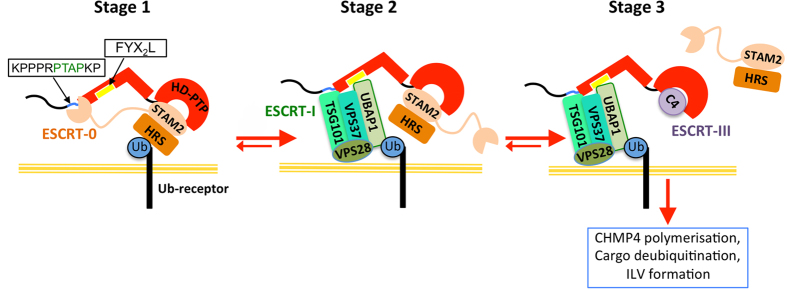
HD-PTP is a scaffold for ESCRT binding. Our model of HD-PTP regulation of ESCRT function involves coordinated binding and displacement of different ESCRTs during endosomal trafficking of ubiquitinated cargo. First, upon internalisation of activated EGFR, ubiquitinated cargo traffics to ESCRT-0. In Stage 1, the ESCRT-0 subunit STAM2 associates with HD-PTP by binding both via its GAT domain (pink oval) at the Bro1 domain, and via its SH3 domain (pink circle) at the PRR (to an SH3 consensus peptide, *PPRPTAPKP*). In Stage 2, STAM2/ESCRT-0 is then released from HD-PTP_PRR_ by the ESCRT-I core subunit TSG101, allowing access of UBAP1 to the CC region and stable binding of ESCRT-I to HD-PTP. UBAP1 binds to the conserved region FYX2 L (yellow) in the CC domain and TSG101 binds to the “PTAP” motif (green) within the SH3 consensus. In Stage 3, binding of ESCRT-III (C4: CHMP4 subunit, purple) to the Bro1 domain of HD-PTP further displaces ESCRT-0, driving the cargo into the MVB pathway. Additional factors, including the presence of deubiquitinating enzymes and ESCRT-III polymerisation, would also drive MVB sorting.

In such a scenario, binding of ESCRT-0 to HD-PTP would prevent binding of ESCRT-I, since occupancy of the STAM2 SH3 domain would completely mask the TSG101 site in the HD-PTP PRR^7^, whilst simultaneous binding of STAM2 to the PRR and the Bro1 domain would likely occlude binding of UBAP1 to the CC domain. Meanwhile, ESCRT-0 would also block CHMP4 from binding to the Bro1 domain, as evidenced by competition between STAM2 and CHMP4^7^ (Fig. 6; stage 1). Release of ESCRT-0 in favour of ESCRT-I and ESCRT-III would then be necessary to drive MVB sorting, and which we speculate could occur by two, possibly simultaneous, competition events. First, the core ESCRT-I subunit TSG101 would bind the PTAP motif in HD-PTP and hence displace the STAM2 SH3 domain. Binding of UBAP1 to the now-available HD-PTP CC domain would further support the engagement of ESCRT-I (Fig. 6, stage 2). Second, binding of CHMP4 would release the GAT domain of STAM2 from the HD-PTP Bro1 domain (Fig. 6, stage 3). Binding affinities of HD-PTP towards STAM2 and CHMP4 are similar^25, 39^. Therefore, progression through this pathway would also depend on environmental factors, including the balance between ubiquitin ligases and deubiquitinating enzymes^7, 49, 50^, alterations in the local concentration of each ESCRT or changes in their abilities to compete with each other, and the polymerisation of CHMP4 (ESCRT-III). Testing this model will be the aim of future work.

## Materials and Methods

### Cloning, protein expression and purification

Four HD-PTP constructs were used in this work: HD-PTP_Bro1-CC_ (1–714) and HD-PTP_Bro1-CC-PRR_ (1–738), cloned into a pET28a vector with restriction sites NdeI and XhoI; HD-PTP_Bro1_ (1–361), cloned into pNIC-28a-Bsa4 (Gift from Opher Gileadi (Addgene #26103); and HD-PTP_CC_ (362–704), cloned into pGEX-4T1 vector using EcoR I and Xho I restriction sites. The GST-tagged FL-CHMP4B construct has been described previously^1^. All constructs were confirmed by DNA sequencing.

All HD-PTP constructs were transformed in BL-21(DE3) *E. coli* cells and protein expression was induced with 0.1 mM IPTG overnight at 20 °C. The His_6_-tagged proteins (HD-PTP_Bro1_, HD-PTP_Bro1-CC_ and HD-PTP_Bro1-CC-PRR_) were purified by metal-affinity column chromatography using Nickel-beads (Qiagen) in 20 mM HEPES pH 7.4, 500 mM NaCl, 10 mM Imidazole and eluted with 250 mM Imidazole, followed by anion-exchange chromatography using a MonoQ 5/50 GL column (GE Healthcare) equilibrated in 20 mM HEPES pH 7.4, 2 mM EDTA, 2 mM DTT and eluted with a gradient of NaCl, and finally by SEC using a Superdex200 column (GE Healthcare) equilibrated with 20 mM HEPES pH 7.4, 0.3 M NaCl, 2 mM EDTA and 2 mM DTT.

GST-tagged HD-PTP_CC_ protein was purified on a 5 ml GSTrap FF column (GE Healthcare) in 20 mM HEPES pH 7.4, 250 mM NaCl, 2 mM DTT buffer and eluted with 20 mM HEPES pH 7.4, 250 mM NaCl, 20 mM reduced glutathione. Fractions containing GST-tagged HD-PTP_CC_ were pooled, and concentrated and buffer-exchanged in 20 mM HEPES pH 7.4, 250 mM NaCl buffer to remove glutathione. The GST-tag was cleaved overnight by thrombin digestion at 4 °C. This digested mixture was passed over a GSTrap FF column, and digested HD-PTP_CC_ was collected in the flow-through. Digested HD-PTP_CC_ was further purified by anion-exchange chromatography using a MonoQ 5/50 GL column and SEC using a Superdex200 column.

FL-CHMP4B was expressed in C41 *E.coli* cells by IPTG induction as above, and purified by lysing bacterial cells in 20 mM HEPES pH 7.4, 250 mM NaCl, 2 mM DTT buffer, followed by sonication and centrifugation at 12,400 × *g* for 1 h. Cleared supernatant was loaded on a GSTrap FF (5 ml) column equilibrated with 20 mM HEPES pH 7.4, 250 mM NaCl, 2 mM DTT, and eluted with 20 mM reduced glutathione in the same buffer.

### Small-angle X-Ray Scattering Analysis

The following concentrations and buffers were used for each sample: HD-PTP_CC_, 2.8 mg/ml in 20 mM HEPES pH 7.4, 300 mM NaCl, 2 mM EDTA and 2 mM DTT; HD-PTP_Bro1-CC_, 12 mg/ml in 50 mM Tris-Cl pH 8.0, 100 mM NaCl, 2 mM EDTA and 2 mM DTT; HD-PTP_Bro1-CC-PRR_, 7.5 mg/ml in 50 mM Tris-Cl pH 8.0, 250 mM NaCl, 2 mM EDTA and 2 mM DTT. SAXS data was collected at beamlines X33 Hamburg DESY (HD-PTP_Bro1-CC_) and BM29 ESRF (HD-PTP_CC_ and HD-PTP_Bro1-CC-PRR_). Data was collected at different concentrations for each sample. We did not observe any aggregation or concentration-dependent effects as shown by the consistency in the calculated *R*_*g*_ values at each concentration (Suppl. Table S1). Data processing was performed with the ATSAS suite^51^ and ScÅtter (http://www.bioisis.net/tutorial/9). The forward scattering *I(*0*)* and the radius of gyration *R*_*g*_ were estimated with PRIMUS^41^ using the Guinier approximation (Suppl. Fig. S1). GNOM^42^ was used to compute the pairwise intra-particle distance distribution function *p*(r) and the maximum distance *D*_*max*_ (Suppl. Fig. S3). Particle shapes were restored *ab initio* using DAMMIN and GASBOR^44^. Twenty simulations were performed and the outputs were averaged and filtered using DAMAVER^52^ to produce the final envelopes (Fig. 2) with a normal spatial discrepancy value of 0.61–0.67 for the DAMMIN models and 1.4–1.8 for the GASBOR models.

### Molecular models and conformational sampling

Libraries of molecular models were generated for each construct as follows. The initial model for HD-PTP_CC_ (residues 362–704) was built from the coordinates of its crystal structure^29^ (PDB ID 5LM2, chain B). Disordered residues from the loop connecting the H1 and H2 helices were rebuilt with standard geometry in CNS^53^. HD-PTP_CC_ conformational variability was explored by varying the distance between the centers of masses of two separate subdomains, CC1 encompassing the “blade” and CC2 encompassing the “shaft” (according to the nomenclature used in ref. 29). A library of conformers was thus generated using torsion angle molecular dynamics (TAMD) as implemented in CNS^53, 54^. For each conformer the fit to the SAXS data was calculated using FoXS^55^, and its hydrodynamic parameters calculated with SOMO^45^.

The initial model for HD-PTP_Bro1-CC_ (residues 1–714) was built from the coordinates of the crystal structure of HD-PTP_Bro1_^38^ (PDB ID 3RAU) and those of the individual HD-PTP_CC_ model with the best fit to the SAXS data (Fig. 2a). Residues connecting the Bro1 and CC domains were rebuilt with standard stereochemistry in CNS. An N-terminal His_6_ tag with the correct sequence and length and was also added to the model. A library of conformers was generated using TAMD to explore different orientations between the Bro1 and CC domains. Different lengths for the linker connecting the Bro1 and CC domains were tried to ensure effective conformational sampling between the two domains. Best results were obtained with a linker spanning residues 360 to 370. Hydrodynamic and dimensional parameters for all the HD-PTP_Bro1-CC_ models were calculated with SOMO and their sedimentation coefficients used to select suitable conformers compatible with the experimentally determined values. The selected pool of models was contrasted to the experimental SAXS profile using FoXS^55^, as previously described^46^.

Models for HD-PTP_Bro1-CC-PRR_ were generated by adding the proximal region of PRR (up to residue 738), to the coordinates of the best HD-PTP_Bro1-CC_ individual model (Fig. 2b). The additional residues were modeled with standard stereochemistry in CNS and their flexible conformation was sampled with TAMD.

### Multi-angle light scattering (MALS) and analytical ultracentrifugation (AUC)

For MALS analyses, samples were injected onto a Superdex-200 10/300 GL column (GE Healthcare) equilibrated with 20 mM Tris-Cl pH 8.0, 100 mM NaCl, 1 mM TCEP (HD-PTP_CC_ and HD-PTP_Bro1-CC_) or 20 mM HEPES pH 7.4, 250 mM NaCl, 2 mM EDTA) (HD-PTP_Bro1-CC-PRR_) and the eluted proteins passed through a Wyatt Helios 18-angle laser photometer with Wyatt EOS QELS detector. Concentrations were measured using a Wyatt rEX differential refractive index detector. Light scattering intensities were measured at different angles relative to the incident beam and data analysis was performed with ASTRA 6 software (Wyatt Technology Corp., CA, USA). Protein fractions from MALS were then used in sedimentation velocity experiments using either Optima XL-I (HD-PTP_CC_) or Optima XL-A (HD-PTP_Bro1-CC_ and HD-PTP_Bro1-CC-PRR_) ultracentrifuges (Beckman Instruments) at 50,000 rpm (18,200 × *g*) at 20 °C and scanning every 60 or 90 seconds respectively, using a wavelength of 280 nm for a total of 200 scans. The sedimentation boundaries were analysed using the program Sedfit v8.7^56^. Solvent corrected sedimentation coefficients (*S*_20,w_), hydrodynamic radii (*R*_*h*_) and frictional ratios (*f/f*_*o*_) were calculated with Sednterp^57^.

### Biosensor Binding studies

Biosensor protein binding studies were performed using the multiplex system ProteOn XPR36 surface plasmon resonance instrument (Bio-Rad Laboratories) in 10 mM HEPES pH 7.4, 150 mM NaCl, 0.05% Tween-20 as running buffer. His_6_-tagged proteins were immobilised on a HTE chip (Bio-Rad Laboratories) at a concentration of 50–100 μg/ml. This gave an immobilization level of proteins typically of 5000–8000 response units (RU). A GLC sensor chip (Bio-Rad Laboratories) was derivatised with anti-GST-antibody (GE Healthcare) using amine coupling, and then used to immobilise FL-CHMP4B at a surface density of 3000–4000 response units (RU).

All experiments were performed at 25 °C. Purified protein HD-PTP_Bro1_, HD-PTP_Bro1-CC-PRR_ and synthetic peptides CHMP4B (^205^KKKEEEDDDMKELENWAGSM^224^) and UBAP1 (^261^SNIKSLSFPKLDSDDSNQKT^280^) (Generon Ltd, UK) were used as analytes in equilibrium binding measurements. Analyte stocks were prepared just prior to the binding experiments and injected (50–100 μl at 100 μl/min) in the horizontal orientation, using serially diluted analyte concentrations chosen to give a suitable spread of responses below and above half-maximal binding. All the binding sensograms were collected, processed and analysed using the integrated ProteOn Manager software (Bio-Rad Laboratories), using the equilibrium binding model: Response = [*A*] * *R*_*max*_/([*A*] + *K*_*D*_) where [*A*] is the analyte concentration and *R*_*max*_ is the maximum response.

### Data Availability

The datasets generated during this study are available from the corresponding author upon reasonable request.

## Electronic supplementary material

Supplementary Material
