# Supplementary material for: The open architecture of HD-PTP phosphatase provides new insights into the mechanism of regulation of ESCRT function
Source: Sci Rep. 2017 Aug 22;7:9151. doi: 10.1038/s41598-017-09467-9 (PMC5567221; doi:10.1038/s41598-017-09467-9)
Supplement: Supplementary file 1 — Supplementary Material [file 41598_2017_9467_MOESM1_ESM.pdf]

Supplementary Information

The open architecture of HD-PTP phosphatase provides new insights into the mechanism of regulation of ESCRT function.

Deepankar Gahloth, Graham Heaven, Thomas A. Jowitt, A. Paul Mould, Jordi Bella, Clair Baldock, Philip Woodman and Lydia Tabernero

Supplementary Table 1. SAXS-derived radii of gyration (R_g) for each protein construct as a function of protein concentration. The data indicate that there were no concentration-dependent effects at the range of concentrations used.

Protein	Conc (mg/ml)	Rg (nm)
HD-PTP$_{CC}$	0.5	4.55
	1.4	4.50
	2.8	4.53
HD-PTP$_{Bro1\text{-}CC}$	6.0	5.58
	12.0	5.55
HD-PTP$_{Bro1\text{-}CC\text{-}PRR}$	1.5	5.82
	2.9	5.78
	7.5	5.83

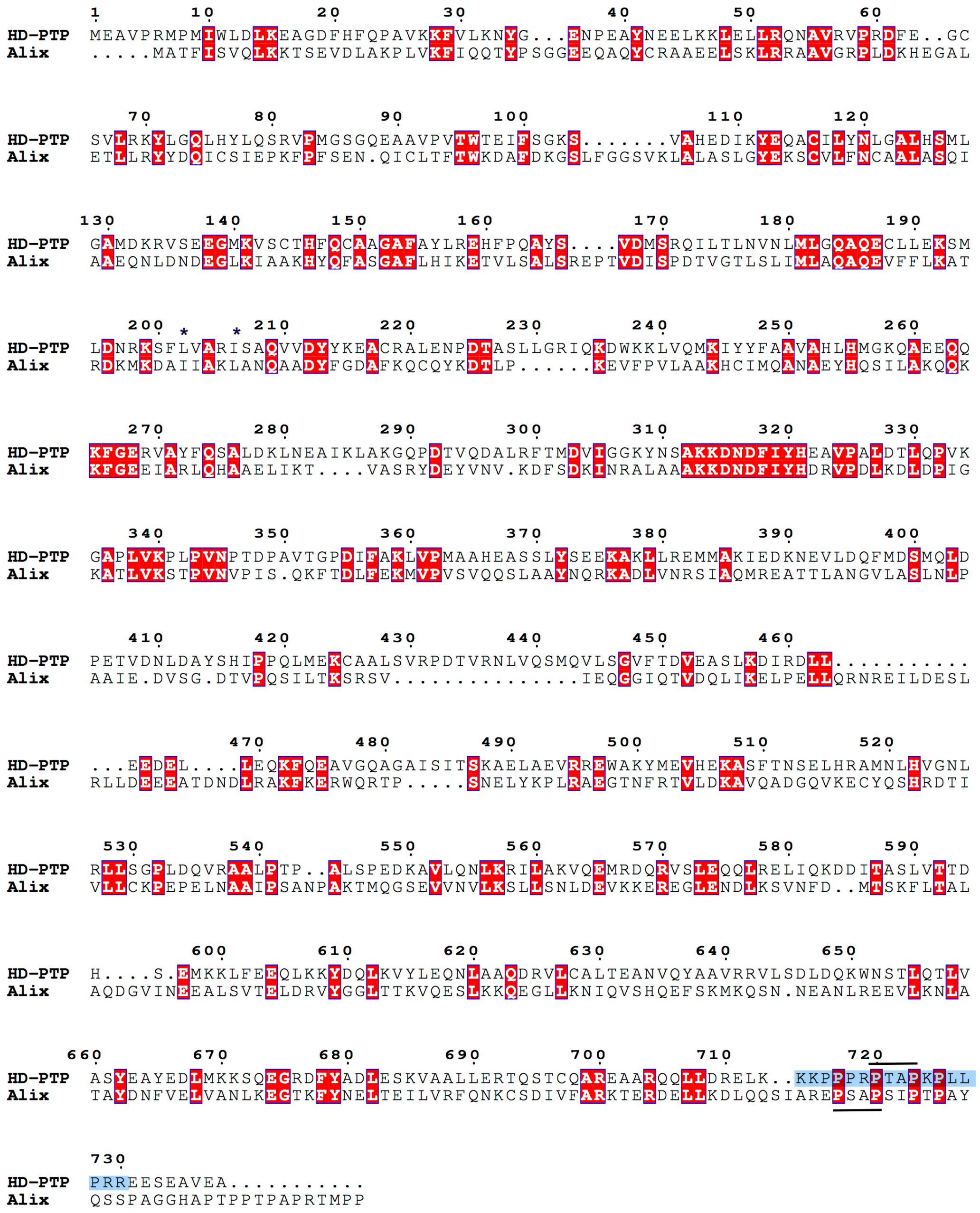
HD-PTP MEAVPRMPMIWLDLKEAGDFHFQPAVKKFVLKNYG...ENPEAYNEELKKLELLRQNAVRVPRDFE..GC
Alix .....MATFISVQLKKTSEVDLAKPLVKFIQQTYPSGGEEQAQYCRAAEELSKLRRAAVGRPLDKHEGAL
HD-PTP SVLRKYLGQLHYLQSRVPMGSGQEAAVPVTWTEIFSGKS.......VAHEDIKYEQACILYNLGALHSML
Alix ETLLRYYDQICSIEPKFPFSEN.QICLTFTWKDAFDKGSLFGGSVKLALASLGYEKSCVLFNCAALASQI
HD-PTP GAMDKRVSEEGMKVSCTHFQCAAGAFAYLREHFPQAYS....VDMSRQILTLNVNLMLGQAQECLLEKSM
Alix AAEQNLDNDEGLKIAAKHYQFASGAFLHIKETVLSALSREPTVDISPDTVGTLSLIMLAQAQEVFFLKAT
HD-PTP LDNRKSFLVARISAQVVDYYKEACRALENPDTASLLGRIQKDWKKLVQMKIYYFAAVAHLHMGKQAEEQQ
Alix RDKMKDAIIAKLANQAADYFGDAFKQCQYKDTLP......KEVFPVLAAKHCIMQANAEYHQSILAKQQK
HD-PTP KFGERVAYFQSALDKLNEAIKLAKGQPDTVQDALRFTMDVIGGKYNSAKKDNDFIYHEAVPALDTLQPVK
Alix KFGEEIARLQHAAELIKT....VASRYDEYVNV.KDFSDKINRALAAAKKDNDFIYHDRVPDLKDLDPIG
HD-PTP GAPLVKPLPVNPTDPAVTGPDIFAKLVPMAAHEASSLYSEEKAKLLREMMAKIEDKNEVLDQFMDSMQLD
Alix KATLVKSTPVNVPIS.QKFTDLFEKMVPVSVQQSLAAYNQRKADLVNRSIAQMREATTLANGVLASLNLP
HD-PTP PETVDNLDAYSHIPPQLMEKCAALSVRPDTVRNLVQSMQVLSGVFTDVEASLKDIRDLL...........
Alix AAIE.DVSG.DTVPQSILTKSRSV...............IEQGGIQTVDQLIKELPELLQRNREILDESL
HD-PTP ...EEDEL....LEQKFQEAVGQAGAISITSKAELAEVRREWAKYMEVHEKASFTNSELHRAMNLHVGNL
Alix RLLDEEEATDNDLRAKFKERWQRTP.....SNELYKPLRAEGTNFRTVLDKAVQADGQVKECYQSHRDTI
HD-PTP RLLSGPLDQVRAALPTP..ALSPEDKAVLQNLKRILAKVQEMRDQRVSLEQQLRELIQKDDITASLVTTD
Alix VLLCKPEPELNAAIPSANPAKTMQGSEVVNVLKSLLSNLDEVKKEREGLENDLKSVNFD..MTSKFLTAL
HD-PTP H....S.EMKKLFEEQLKKYDQLKVYLEQNLAAQDRVLCALTEANVQYAAVRRVLSDLDQKWNSTLQTLV
Alix AQDGVINEEALSVTELDRVYGGLTTKVQESLKKQEGLLKNIQVSHQEFSKMKQSN.NEANLREEVLKNLA
HD-PTP ASYEAYEDLMKKSQEGRDFYADLESKVAALLERTQSTCQARREAARQQLLDRELK..KKPPPRPTAPKPLL
Alix TAYDNFVELVANLKEGTKFYNELTEILVRFQNKCSDIVFARKTERDELLKDLQQSIAREPSAPSIPTPAY
HD-PTP PRREESEAVEA...........
Alix QSSPAGGHAPTPPTPAPRTMPP

Supplementary Figure 1. Sequence alignment of HD-$PTP_{Bro-CC-PRR}$ (1-738) with Alix (1-747). Conserved residues are highlighted in red. CHMP4 binding residues are marked with * and the STAM-SH3 binding site [7] is highlighted in blue. The TSG101 binding PTAP motif is marked with a black underline. The corresponding PSAP motif in Alix is also marked.

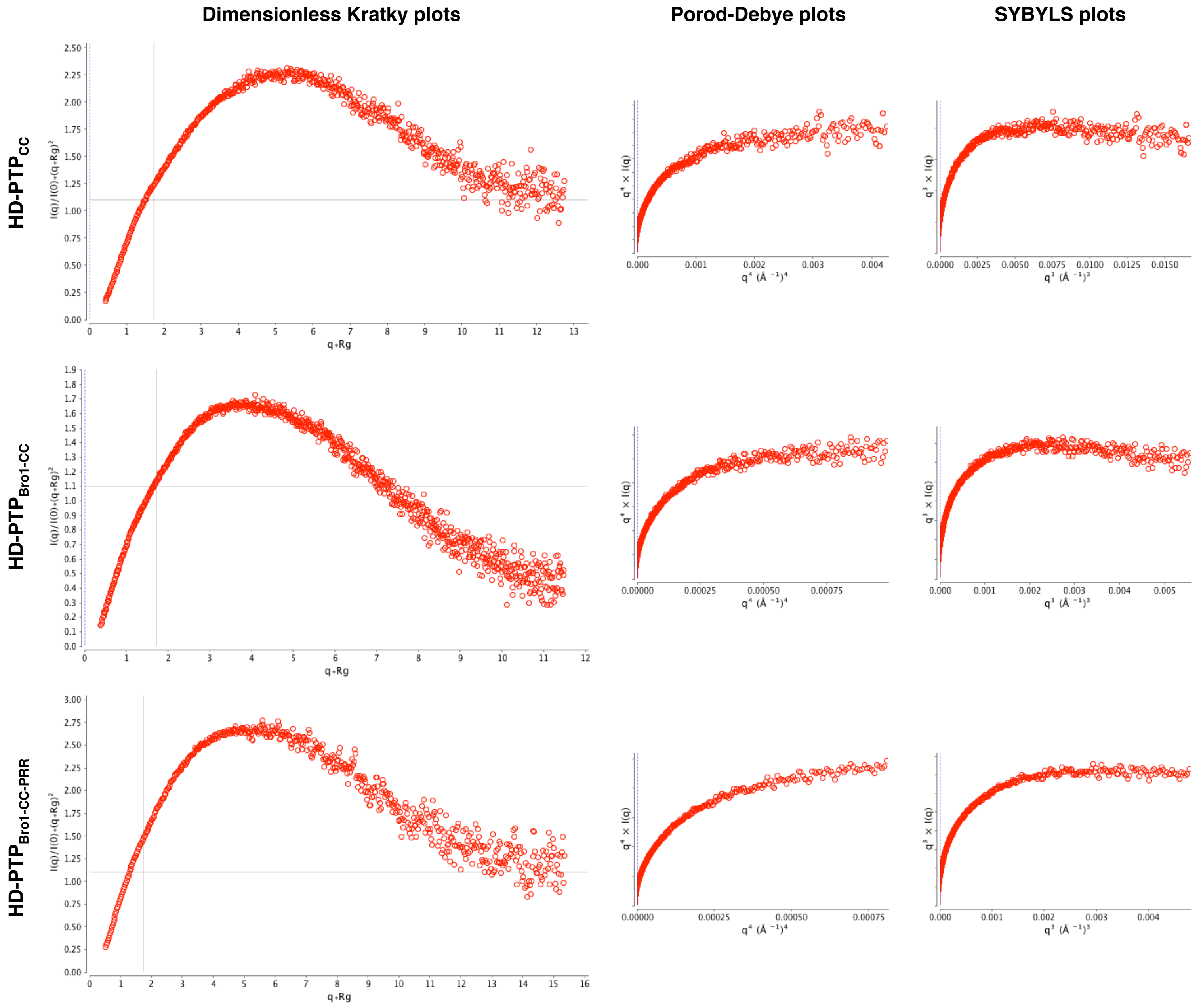
Dimensionless Kratky plots
Porod-Debye plots
SYBYLS plots
HD-PTP$_{CC}$
HD-PTP$_{Bro1\text{-}CC}$
HD-PTP$_{Bro1\text{-}CC\text{-}PRR}$
I(q)/I(0)*(q*Rg)^2
q*Rg
q^4 × I(q)
q^4 (Å^{-1})^4
q^3 × I(q)
q^3 (Å^{-1})^3

Supplementary Figure 2. SAXS Analysis of shape and flexibility for HD-PTP$_{CC}$ (top), HD-PTP$_{Bro1\text{-}CC}$ (middle) and HD-PTP$_{Bro1\text{-}CC\text{-}PRR}$ (bottom). Dimensionless Kratky plots (left column) show $\mathbf{I(q)/I(0) \times (q \times \boldsymbol{R_g})^2}$ against $\mathbf{q \times \boldsymbol{R_g}}$ where $\mathbf{I(q)/I(0)}$ is the normalised X-ray scattering intensity, $\boldsymbol{R_g}$ is the radius of gyration in Å and **q** is the modulus of the scattering vector in $Å^{-1}$. The cross hairs indicate the globularity point (at $q \times R_g = \sqrt{3}$, with a magnitude of $3e^{-1}$). Globular compact particles should show a peak at this position irrespective of size, composition and concentration, whereas intrinsically disordered proteins would show a hyperbolic plateau [46]. All three constructs show a peak shifted to the right and then drop back towards zero, suggesting folded but elongated proteins. The Porod-Debye plots (middle column) show $\mathbf{q^4 \times I(q)}$ against $\mathbf{q^4}$ whereas SIBYLS plots (right column) show $\mathbf{q^3 \times I(q)}$ against $\mathbf{q^3}$, where **I(q)** is the X-ray intensity and **q** is the modulus of the scattering vector in $Å^{-1}$. Data reaching a plateau at high q^4 values in the Porod-Debye plot would indicate compact particles, whereas a pronounced positive slope in the Porod-Debye plot and a plateau in the SIBYL plot would suggest particles with large flexibility (46). The HD-PTP$_{CC}$ and HD-PTP$_{Bro1\text{-}CC}$ plots are consistent with compact particles in solution and only the HD-PTP$_{Bro1\text{-}CC\text{-}PRR}$ construct show some sign of limited flexibility due to the presence of the unstructured PRR region. Graphs prepared using ScÅtter (http://www.bioisis.net/tutorial/9).

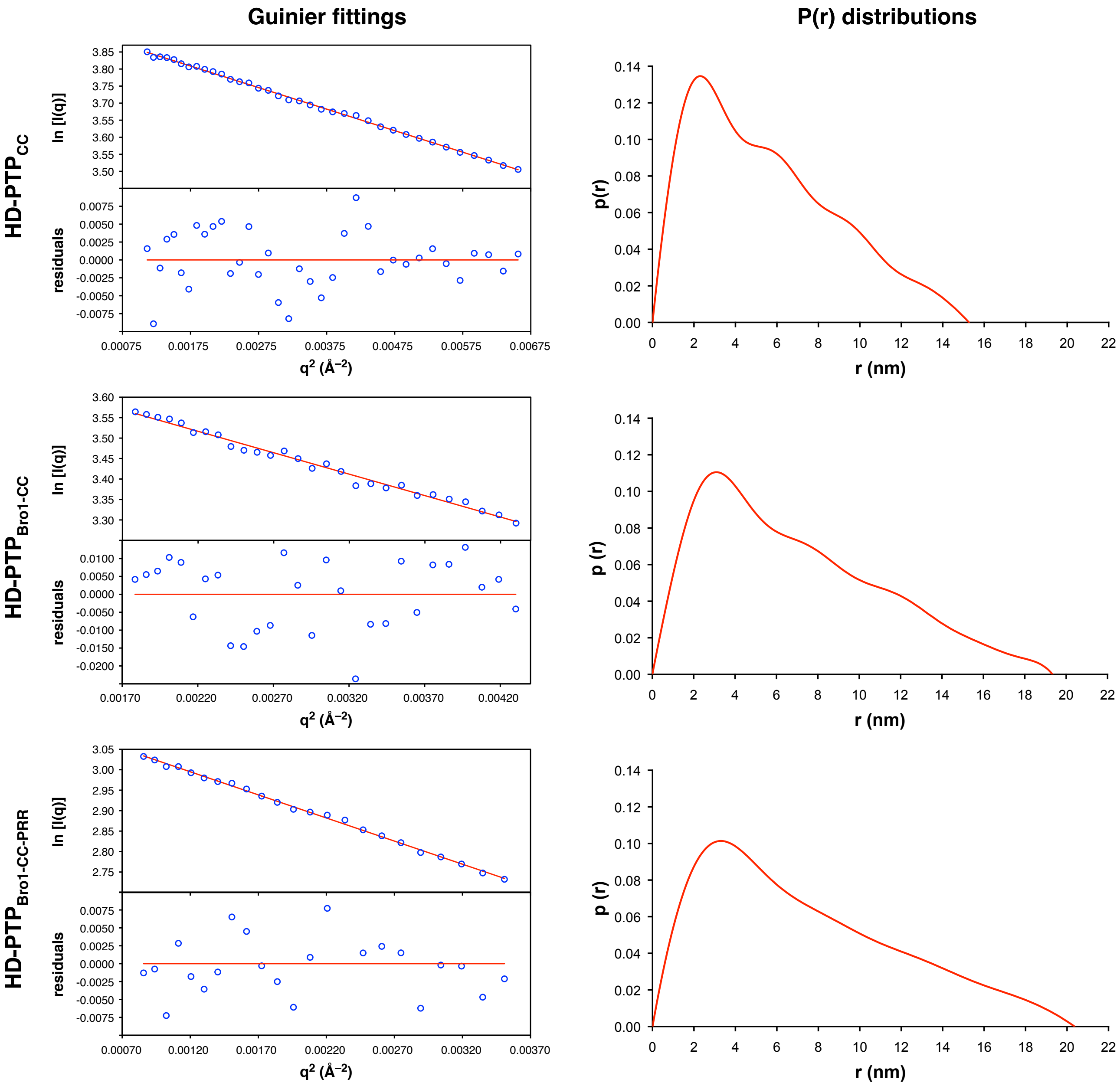
Guinier fittings
P(r) distributions
HD-PTP$_{CC}$
HD-PTP$_{Bro1-CC}$
HD-PTP$_{Bro1-CC-PRR}$
ln [I(q)]
residuals
q^2 (Å^{-2})
p(r)
r (nm)

Supplementary Figure 3. Guinier fitting plots (left column) show the logarithm of the X-ray intensity **ln [I(q)]** as a function of the square of the modulus of the scattering vector $\mathbf{q^2}$ (in Å^{-2}) for each construct. The radii of gyration (R_g) measured from the slopes of the linear regions at low values of q^2 are: 4.53 nm for HD-PTP$_{CC}$, 5.55 nm for HD-PTP$_{Bro1\text{-}CC}$ and 5.83 nm for HD-PTP$_{Bro1\text{-}CC\text{-}PRR}$. Experimental data are shown as blue empty circles and the lines of best fit are show in red. Residual plots show random distributions of differences between experimental values and those calculated from the lines of best fit. P(r) distribution plots (right column) show the probability of distance between scatterers **p(r)** against the distance **r** (in nm) for each construct. The maximum inter-scatterer distances D_{max} are, respectively, 15.3 nm for HD-PTP$_{CC}$, 19.3 nm for HD-PTP$_{Bro1\text{-}CC}$ and 20.4 nm for HD-PTP$_{Bro1\text{-}CC\text{-}PRR}$. Each P(r) distribution plot has been normalised so that the area under the curve equals 1.
